# Infliximab for Peripheral Ulcerative Keratitis Treatment

**DOI:** 10.1097/MD.0000000000000176

**Published:** 2014-12-05

**Authors:** Valentín Huerva, Francisco J. Ascaso, Andrzej Grzybowski

**Affiliations:** From the Department of Ophthalmology (VH), University Hospital Arnau de Vilanova; IRB Lleida (VH), Lleida; Department of Ophthalmology, “Lozano Blesa” University Clinic Hospital (FJA); Instituto Aragonés de Ciencias de la Salud (FJA), Zaragoza, Spain; and Department of Ophthalmology (AG), Poznań City Hospital, Poznań, Poland.

## Abstract

Biologic agents such as anti-TNFα have been employed in treatment paradigms for ocular inflammation. Peripheral corneal ulceration (PUK) is a devastating disorder consisting of a crescent-shaped area of destructive inflammation at the margin of the corneal stroma. It is associated with an epithelial defect, the presence of stromal inflammatory cells, and progressive stromal degradation and thinning, leading to ocular perforation and devastating visual loss. Macroulcerative PUK is usually a local manifestation of a systemic vasculitis. In many cases, the disease may be resistant to high doses of systemic corticosteroids and immunosuppressants. Chimeric anti-TNFα has been employed when all other treatments have failed. Isolated cases and short series of cases have been reported. This paper summarizes the available reports on the use, efficacy, and safety of infliximab in the treatment of PUK.

## INTRODUCTION

Peripheral corneal ulceration is a potentially devastating disorder, consisting of a crescent-shaped region of destructive inflammation at the margin of the corneal stroma, associated with an epithelial defect, the presence of stromal inflammatory cells, and progressive stromal degradation and thinning. Commonly referred to as peripheral ulcerative keratitis (PUK), it can quickly initiate progressive necrosis of the corneal stroma, leading to perforation and blindness. Ocular causes, such as infection, eyelid malposition, lagophthalmos, or neurotrophic defects may produce PUK.^1–3^ However, macroulcerative peripheral keratitis is usually a local manifestation of a systemic vasculitis. The most common type of PUK caused by local ocular autoimmunity is the Mooren ulcer. Table 1 shows the major autoimmune diseases that may produce PUK. For this reason, control of this disease requires systemic rather than local treatment.^2^ Local treatment requires the suppression of any ulceration, the provision of tectonic support, and the facilitation of wound healing.^2^ However, local treatment of PUK has, in some situations, been unsuccessful.^1,2,4^ In many cases, the disease may be resistant to high doses of systemic corticosteroids and immunosuppressants.^2–4^ When a corneal perforation occurs, procedures employing cyanoacrylate glue, conjunctival flap, lamellar patch flap, or penetrating keratoplasty may be necessary.^1,2^ If the disease has progressed to a point requiring this level of intervention, the visual prognosis may be very compromised. New systemic treatments, such as rituximab, have been successfully employed in recent years in the treatment of PUK.^4–6^ Additional reports have appeared describing the utility of infliximab in PUK treatment. The majority of these are isolated case reports. The clinical impact of neutralizing tumor necrosis factor alpha (TNFα) activity in inflammatory diseases has been likened to that of corticosteroids. This comparison highlights the revolutionary impact that anti-TNFα agents have had in the treatment of chronic inflammatory disorders. Randomized controlled trials have proven the efficacy of anti-TNFα agents in the treatment of rheumatoid arthritis, juvenile idiopathic arthritis, ankylosing spondylitis, psoriatic arthritis, and fistulizing Crohn disease, and the clinical use of TNFα-targeted therapies in these diseases is now widespread.^7^ Infliximab, the most commonly used agent, is a chimeric monoclonal antibody composed of the variable region of a mouse antibody joined to the constant region of human IgG1. Infliximab binds with high affinity to both soluble and transmembrane forms of TNFα.^8^ TNFα is bound rapidly and irreversibly, and when infliximab is present in excess it can block all 3 receptor-binding sites on TNFα.

**TABLE 1:**
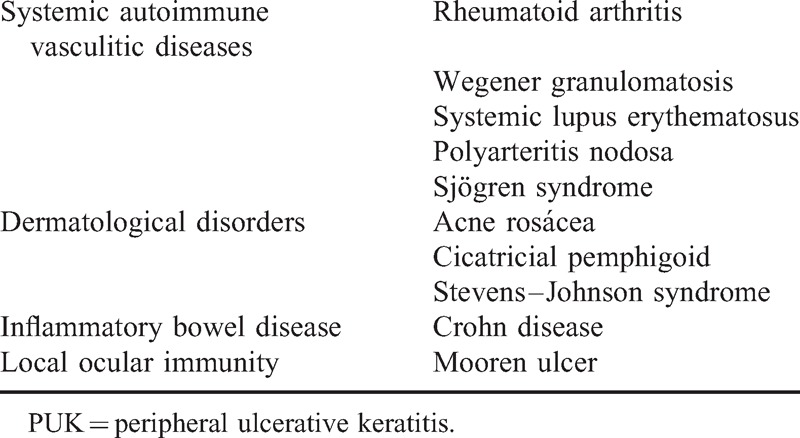
Major Autoimmune Diseases Causing PUK

The aim of this study was to review the published cases of infliximab administration for resistant cases of PUK in different clinical situations and to establish whether it may be a good alternative treatment in such cases.

## MATERIALS AND METHODS

A literature search was performed, using PubMed and Google Scholar. The keywords used were peripheral ulcerative keratitis treatment, PUK treatment, biologic agents and PUK, anti-TNFα and PUK, and infliximab and PUK. References cited in the identified reports were also reviewed. Peer-reviewed meeting abstracts were also considered. From the articles identified, the following data were obtained: type of associated systemic disease, prior systemic therapies employed, infliximab response, and outcome after therapy. Cases in which 2 eyes were affected were considered as 1 case. Causes of treatment cessation were also considered.

## RESULTS

Table 2 shows the cases successfully treated with infliximab. Local surgical treatments for PUK using amniotic membrane or sectoral keratoplasty have not been specifically identified in the table. A total of 22 patients from 12 reports have been included.^9–20^ Many cases had required several previous interventions because of ocular perforation, prior to the use of infliximab.^16^ In the majority of cases, infliximab was administered in response to either corneal perforation or imminent corneal perforation because of severe inflammation and corneal thinning, despite previous systemic anti-inflammatory or immunosuppressive treatment, and a tectonic surgical procedure was necessary. Despite local repair of PUK and systemic immunosuppressive treatment, corneal thinning and perforation reoccurred in several cases.^9–11,14,16–19^ In most cases, the authors administered infliximab after institutional authorization because of the failure of all previous therapeutic agents employed. Infliximab was not used as a first-line therapy in any of the reported cases. Of the 22 cases examined, rituximab administration was necessary for the control of inflammation in 2 cases that were secondary to rheumatoid arthritis.^12^ In another case, where an association with rheumatoid arthritis and Crohn disease was present, a suboptimal resolution was observed, and additional subcutaneous methotrexate was required for resolution of the inflammation.^13^ In another, infliximab was discontinued because of the return of scleritis symptoms.^19^ Despite a good initial response in another case, an increase in the dose and frequency of administration became necessary when a corneal perforation occurred during treatment.^14^ In the remaining 17 patients (77.27%), no further keratolysis after receiving infliximab was reported. The frequency of infusions used varied among the case studies. In some, treatment had to be interrupted because of the appearance of systemic or local side effects, including intensive care unit hospitalization for pneumonia,^19^ deep venous thrombosis,^14^ or the return of scleritis symptoms.^19^

**TABLE 2:**
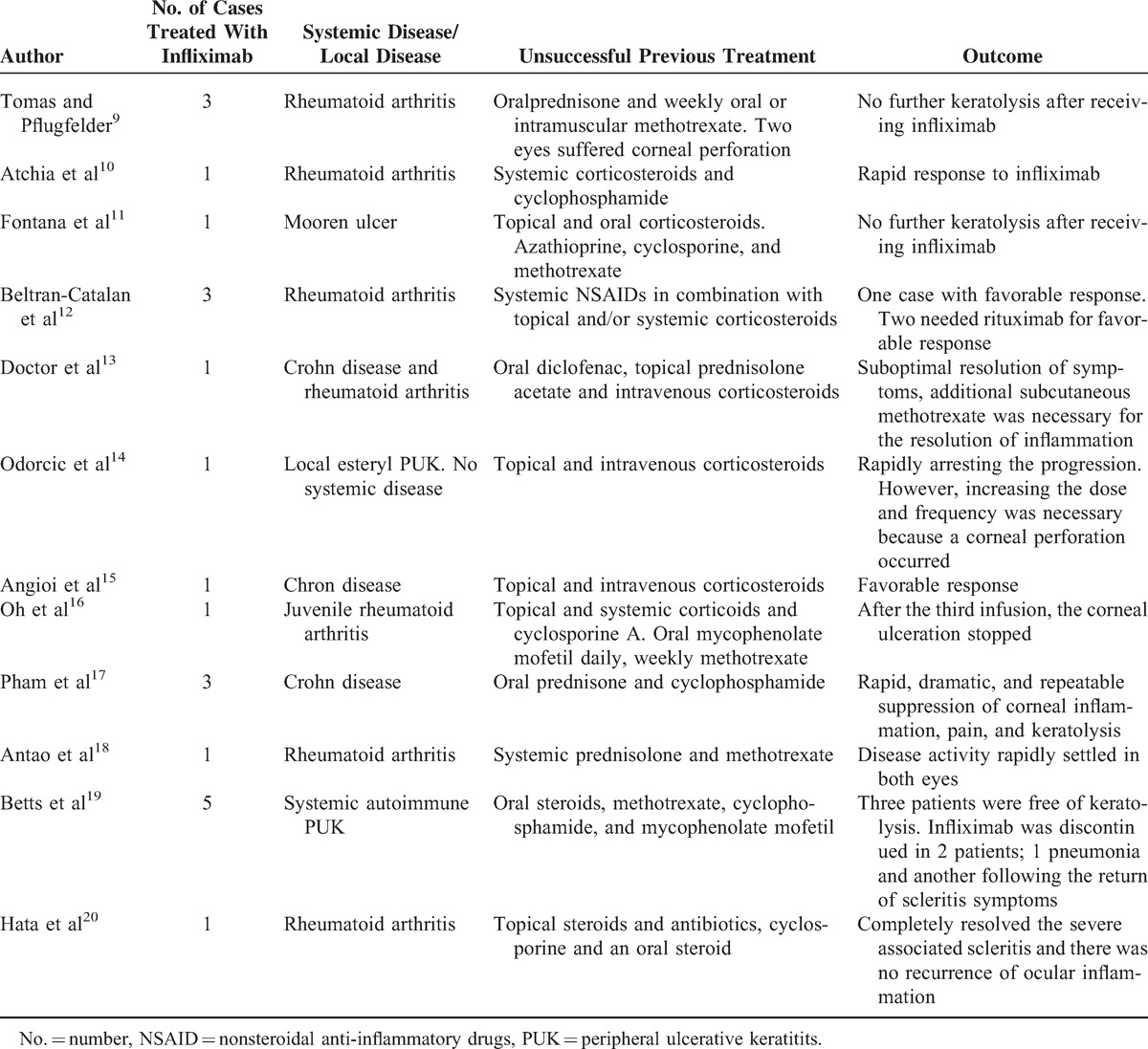
Published Cases of PUK Treated With Infliximab Infusion

## DISCUSSION

Infliximab (Remicade; Centocor Ortho Biotech Inc, Horsham, PA) was approved for use by the US Food and Drug Administration in 1999. Use of infliximab for ocular inflammation was first reported in 2001 for patients with panuveitis and rheumatoid arthritis-associated scleritis.^21,22^ It is currently indicated for the treatment of connective tissue or vasculitic autoimmune diseases that may accompany PUK, as well as for other ocular inflammatory states, such as necrotizing scleritis and uveitis. Infliximab is a specific, chimeric monoclonal antibody, directed against the proinflammatory cytokine TNFα, which stimulates production of the matrix metalloproteinases responsible for corneal stromal lysis in PUK. It binds both soluble and transmembrane TNFα by blocking its receptor binding. Cells expressing transmembrane TNFα bound to infliximab may also be susceptible to complement-mediated lysis, potentially increasing its anti-inflammatory effect.^3^ The dosage of infliximab varies from 3 mg/kg intravenously for rheumatoid arthritis to 5 mg/kg intravenously for Crohn disease,^3,17^ and it is administered at weeks 0, 2, and 6, and then every 8 weeks for up to 18 months. Improvement may occur 1 to 2 weeks after the first infusion. The optimal frequency and dosing of infliximab for PUK is not clear, because of the different conditions that may produce this corneal disease, the different previous and/or concomitant immunosuppressants that may be employed, and the possible systemic side effects that may lead to discontinuation of treatment.

All the reported cases used different numbers of infusions. Treatment was stopped when a systemic effect appeared. However, remission of the disease was maintained during the follow-up period. Congestive heart failure is an absolute contraindication for administering infliximab. Also, opportunistic infections such as tuberculosis must be eliminated prior to treatment. Serious side effects including myocardial infarction, pulmonary embolus, deep venous thrombosis, and retinal vein occlusion have been reported.^23,24^

Because of the severity of these potential side effects, infliximab is not indicated in the first instance for PUK treatment. However, in severe cases where all previous treatments have failed, the literature suggests that it may be a valuable elective option. In 77.27% of cases, keratolysis was halted. Controlling this destructive process secondary to inflammation can preserve both the integrity of the eyeball and its vision.

Adalimumab (Humira; Abbott Pharmaceutical Inc) also is actually considered a valid option for inflammatory eye disorders. Adalimumab is a fully humanized recombinant IgG1 monoclonal antibody specific for TNFα. Pharmacokinetics of this agent results in a sustained neutralization of TNFα in a manner similar to that of infliximab. However, the subcutaneous delivery method results in smooth and uniform concentration time profiles at steady state.^25^ Adalimumab has shown effectiveness in treating several systemic autoimmune diseases such as rheumatoid arthritis, ankylosing spondylitis, and psoriatic arthritis. Adalimumab also has been employed successfully in chronic refractory cases of intraocular inflammation.^26–28^ Moreover, adalimumab showed good safety and efficacy profiles in ocular inflammation use including refractory noninfectious childhood chronic uveitis.^27,28^ Its use has been associated with improvement in visual acuity and improving or stable intraocular inflammation. However, systemic or periocular steroid treatment continuation is necessary.^27,28^ Unlike other anti-TNFα agents, adalimumab may offer several advantages such as easier administration and better patient compliance. Lower rate of adverse events may be posible.^26^ At present, adalimumab is a promising drug for the therapy of refractory uveitis.^26^ However, there are no studies on its use in the treatment of the PUK.

## CONCLUSIONS

The last decade has seen an increase in reports of infliximab use in severe PUK, demonstrating its effectiveness in modulating inflammation when all previous treatments have failed. Systemic administration of this chimeric anti-TNFα halted the destruction of corneal tissue, whereas preserving the integrity of the eye in most of the cases reported. Randomized studies are necessary to establish if this treatment may be used as a primary therapy in severe cases of PUK. Additional studies, including different anti-TNFα antibodies, such as adalimumab, are necessary to establish the effectiveness and safety of this treatment in severe PUK.
